# Estimating the Relative Vaccine Efficacy of mRNA-1010 to High-Dose Influenza Vaccine in Adults ≥65 Years Using a Correlate of Protection Framework

**DOI:** 10.3390/vaccines14090750

**Published:** 2026-08-28

**Authors:** Van Hung Nguyen, Ethan Settembre, John Youhanna, Ekkehard Beck, Nicolas Van de Velde, Keya Joshi

**Affiliations:** 1 VHN Consulting Inc., Montreal, QC H2V 3L8, Canada; 2 Moderna Inc., 325 Binney Street, Cambridge, MA 02142, USA

**Keywords:** influenza, mRNA-1010, high-dose inactivated influenza, correlate of protection, relative vaccine efficacy, older adults

## Abstract

Background/objective: Older adults experience a disproportionate burden of severe influenza. In the P303 trial (NCT058279789), mRNA-1010 showed greater immunogenicity versus high-dose inactivated influenza vaccine (HD-IIV4) in adults ≥65 years of age. Here, we used a correlate-of-protection framework to estimate the relative VE (rVE) of mRNA-1010 compared with HD-IIV4. Methods: A sigmoidal function was used to model the relationship between geometric mean haemagglutinin assay inhibition (HAI) titers (GMTs) and vaccine efficacy (VE) using data from the P304 trial (NCT06602024), which compared mRNA-1010 and the standard-dose IIV4 in adults ≥50 years of age. The model was then applied to GMTs from the P303 trial to estimate rVE of mRNA-1010 vs. HD-IIV4 in adults ≥65 years. Temporal waning and weighting of case distributions were incorporated to provide a season-integrated estimate. Simulations were performed for three influenza seasons (A/H1N1-dominant, A/H3N2-dominant, and balanced), with a sensitivity analysis varying vaccination and epidemic peak timing. Results: At Day 29, rVE for mRNA-1010 vs. HD-IIV4 was 24.1% (95% CI: 18.2–29.5%) against A/H1N1, 14.5% (8.3–20.3%) against A/H3N2, and 21.0% (16.0–25.8%) against B/Victoria. The season-integrated rVE was 12.4% (3.4–21.3%) across all three strains, with point estimates ranging from 11.6% to 12.6%. Similar findings were seen across simulated seasons, with rVE estimates ranging from 8.8% to 14.3% when varying vaccination timing, peak timing, and strain dominance. Conclusions: Based on this CoP framework, mRNA-1010 was projected to have higher VE than HD-IIV4 in adults ≥65 years of age across a range of simulated epidemiological conditions.

## 1. Introduction

Globally, seasonal influenza viruses cause an estimated 3 to 5 million cases of severe illness and up to 650,000 deaths each year [1]. Older adults (≥65 years of age) experience the highest burden of severe influenza outcomes. In the 2024–2025 season in the US, while only 8.8% of influenza-related symptomatic cases occurred in adults ≥65 years of age, 70.6% of influenza-related deaths and 57.4% of influenza-related hospitalizations occurred in this age group, with a cumulative hospitalization rate of 666.3 per 100,000 [2]. While the burden of influenza varies per season, older adults consistently experience the most cases of severe disease, accounting for 45–72% of hospitalizations and 66–87% of deaths across seasons from 2010 to 2025. Infection with influenza can also lead to serious cardiovascular complications in older adults in particular, with a 4-fold higher risk of myocardial infarction and 5-fold higher risk of stroke within one month post-infection, as well as one in seven influenza infections resulting in an acute cardiac event in this age group [3,4].

One of the main drivers of the disproportionate burden of severe influenza in older adults is immunosenescence, the age-related decline in immune system function. Immunosenescence reduces the effectiveness of immune responses to infection, increasing the risks of more severe disease manifestations and related complications [5,6]. In addition, immunosenescence reduces vaccine responses, leaving individuals more vulnerable to infection despite vaccination [7,8]. Variable influenza vaccine effectiveness (VE) on a season-by-season basis compounds this risk, with multiple factors contributing to the potential for poor vaccine performance. World Health Organization (WHO)-recommended vaccine virus strains are announced months before the start of each influenza season to allow sufficient time for vaccine manufacture. As influenza viruses are continually evolving through antigenic drift, this time lag results in the potential for mismatch between the circulating and vaccine strains, reducing VE [9]. In addition, most seasonal influenza vaccines are still produced using the traditional method of growth in eggs, with egg adaptation of virus strains (particularly A/H3N2) further contributing to potential vaccine mismatch [10,11,12]. Antigenic drift or egg adaptation potentially affected seasonal influenza VE in all but one of the Northern Hemisphere influenza seasons between 2011 and 2020 [9], highlighting the unmet need for vaccines that reduce the risk of mismatch.

mRNA vaccine technology has multiple advantages over traditional egg-based vaccines, with the potential for a rapid manufacturing time, reducing the lag between decisions on strain content and availability of the vaccine, no reliance on eggs, and the potential for flexible antigenic composition, including multiple component targets [9]. mRNA-1010 (mFLUSIVA, Moderna) is an mRNA-based seasonal influenza vaccine recently approved by the US Food and Drug Administration [13] that encodes hemagglutinin glycoproteins from the WHO-recommended cell- or recombinant-based reference influenza strains. In the P304 phase 3 trial (NCT06602024), which enrolled 40,703 adults aged 50 years and older across 301 sites in 11 countries during the 2024–2025 Northern Hemisphere influenza season, the relative vaccine efficacy (rVE) of mRNA-1010 versus standard-dose quadrivalent inactivated influenza vaccine (SD-IIV4) was 26.6% (95% CI: 16.7% to 35.4%), meeting the prespecified criteria for non-inferiority, superiority, and higher-level superiority. Consistent findings were seen in adults ≥65 years of age, where the overall rVE was 27.4% (12.1% to 40.0%). In an exploratory analysis per-strain rVE estimates were 29.6% (16.4% to 40.7%) against A/H1N1, 22.2% (4.3% to 36.9%) against A/H3N2, and 29.1% (−18.5% to 57.5%) against B/Victoria [14].

In many countries, enhanced (adjuvanted or high-dose [HD]) vaccines are preferentially recommended for the vaccination of older adults to improve vaccine responses, with both types of vaccine associated with similar levels of effectiveness in this age group, and vaccination recommendations do not distinguish between them [15,16,17]. In the three-part P303 trial (NCT058279789), mRNA-1010 showed consistently higher GMTs than HD-IIV4 across all three strains, with geometric mean ratios of 1.2 to 1.3 in adults ≥65 years of age [18]. This is the only phase III randomized control trial that compared a candidate seasonal influenza vaccine to an Advisory Committee on Immunization Practices (ACIP) preferentially recommended enhanced vaccine. While the P304 trial showed statistically significantly superior rVE compared with SD-IIV4 in older adults, no direct head-to-head studies have evaluated rVE of mRNA-1010 compared with enhanced vaccines. Using correlates of protection (CoP) is an established method for estimating VE in populations where direct efficacy estimates are not available (e.g., different age groups). In this analysis, we use existing validated approaches to develop a CoP model to estimate the rVE of mRNA-1010 compared with HD-IIV4 in the prevention of laboratory-confirmed influenza. VE denotes the modelled probability of protection against laboratory-confirmed influenza derived from the titer–protection relationship, and rVE denotes the corresponding proportional difference between two vaccines. The model uses rVE estimates from the P304 trial combined with haemagglutinin inhibition assay (HAI) titers from the P303 trial, as well as the impacts of waning VE post-vaccination and potential differences across influenza seasons. In addition, we translate the rVE between the two vaccines into public health impact metrics, using a coverage equivalence framework.

## 2. Methods

### 2.1. Model Design and Parameters

The initial CoP model was based on the sigmoidal model described by Dunning et al. [19], which was used to estimate VE at Day 29. The model estimated the probability of protection at any given HAI titer (see Equation (S1) in the Supplementary Materials). The midpoint of the sigmoidal curve, N50 (i.e., the HAI titer at which the probability of protection is 50%), was set at 40 for all strains, age groups, and analyses, in line with data from previous studies and the seroprotection threshold adopted in the FDA and EMA guidance for the immunogenicity evaluation of influenza vaccines [19,20,21]. N50 and β characterize the relationship between titer and protection, which is a property of the antibody–virus interaction rather than of the host; both were therefore held constant across age groups, with population differences entering the model through the observed titer distributions. Group-level aggregate GMTs [22] and associated 95% confidence intervals from the P304 trial were used for estimation of individual slope parameters (β) for each influenza vaccine strain, resulting in values of 1.1425 for A/H1N1, 1.0437 for A/H3N2, and 1.1504 for B/Victoria. β was calibrated per strain via bisection on P304 data: the parameter was iteratively adjusted until the model’s projected season-integrated rVE (Cox hazard ratio formula) reproduced the observed P304 rVE for that strain (26.6% overall; 29.6%, 22.2%, and 29.1% for A/H1N1, A/H3N2, and B/Victoria, respectively). The calibrated β values are therefore specific to the mRNA-1010 trial context and are anchored to P304’s aggregate GMTs and case distribution, rather than representing independently estimated biological constants. The projected incremental VE of mRNA-1010 and HD-IIV4 was then calculated at Day 29 as the fraction of preventable cases actually prevented by vaccination, relative to pre-existing baseline immunity (Equation (S2) in Supplementary Materials).

To incorporate the effects of waning VE throughout the influenza season, a linear waning model as described by Ferdinands et al. [23] was applied symmetrically to both treatment arms for timepoints post-Day 29, with a 7% reduction in VE per month (λ), based on published intraseason estimates from 2011–2012 to 2014–2015 in the US [23] (Equation (S3) in Supplementary Materials). Temporal variability in case distribution was accounted for by weighting based on the total number of cases reported in each month in the P304 trial (Equation (S4) in Supplementary Materials). These two steps were applied at the calibration stage, where the integrated VE estimates were used to reproduce the observed P304 rVE as 1 − (1 − VE_mRNA_1010)/(1 − VE_comparator). The 95% confidence intervals of the rVE estimates were calculated using a parametric bootstrap procedure with 5000 replicates.

Because β is fitted by construction to reproduce P304’s observed rVE, close replication at this stage confirms the numerical convergence of the calibration rather than independent model validation. The critical assumption underlying this analysis, as used in previous analyses estimating VE for influenza vaccines from HAI titers [24], is that the calibrated sigmoidal relationship between HAI titer and protection is transportable from the P304 population to the P303 population, before being applied to project rVE of mRNA-1010 versus HD-IIV4. In both trials, HAI titers for all treatment arms were measured against the same panel of cell-derived viruses, corresponding to the cell-based strains recommended by the WHO for the relevant season: A/Wisconsin/67/2022, A/Darwin/6/2021 and B/Connecticut/01/2021 (a B/Austria/1359417/2021-like virus) in P303 Part C, and A/Wisconsin/67/2022, A/Massachusetts/18/2022 and B/Connecticut/01/2021 in P304 (Moderna, data on file). This assay format was selected so that the test reagents would correspond as closely as possible to circulating influenza viruses, which replicate in mammalian hosts, whereas propagation in eggs can introduce adaptive changes that alter HA antigenicity relative to circulating strains. As both arms within each trial were titrated against the same antigen panel, the choice of reference antigen sets the scale of the measured titers without altering the relationship between titer and protection.

### 2.2. Objectives

#### 2.2.1. rVE at Day 29 and Effects of Waning

The calibrated model was applied to HAI GMT data from the P303 trial to estimate rVE of mRNA-1010 vs. HD-IIV4 in adults ≥65 years of age at Day 29 (peak immunogenicity). (Supplementary Table S1 For each vaccine arm and influenza strain, an exponential decay curve was fitted through the two observed P303 Part C GMT time points (Day 29 and Day 181): GMT(t) = GMT_D29 × exp(−λt), where λ = −ln(GMT_D181/GMT_D29)/Δt (Δt = 4.99 months). This produced a continuous, arm- and strain-specific GMT trajectory, which was passed through the calibrated sigmoidal CoP model at monthly intervals to generate a continuous VE trajectory from Day 29 to Day 181, following Dunning and Coudeville [19,25]. Waning in the projection is therefore derived from the observed GMT trajectories rather than from the linear rate used at calibration. This step was necessary because the influenza epidemic typically peaks 2 to 4 months post-vaccination, whereas the VE at Day 29 alone reflects only peak immunogenicity and overstates protection during the period of highest viral circulation. The same exponential projection was applied separately to the lower and upper bounds of the 95% CI at each time point, producing arm-specific bounding trajectories from which the 95% CI of the VE and rVE could be derived at any monthly time point. Using these bounding trajectories, the CI overlap onset, i.e., the earliest time point at which the lower-bound trajectory of mRNA-1010 fell below the upper-bound trajectory of HD-IIV4, was identified for each strain, marking the point beyond which mRNA-1010 no longer demonstrated a statistically significant advantage over HD-IIV4. A decision framework was then applied to evaluate the clinical implications of the CI overlap beyond this point, based on four criteria: 1. whether CI overlap occurred (yes or no), 2. VE during the period of CI overlap (from point of first overlap until end of follow-up), 3. rVE during the period of overlap, 4. GMR during the period of overlap, with decision rules based on the definitions outlined in Supplementary Table S2.

No minimum clinically important difference or equivalence margin was prespecified; these categories are descriptive, and the inference on the difference between the two vaccines is based on the confidence interval of the rVE itself. The estimation of the rVE of the two vaccines across the entire influenza season (season-integrated rVE) was performed by combining the monthly estimated VE trajectories weighted by the average contribution of each time point to the total seasonal case burden (Supplementary Table S3, see Supplementary Materials Equation (S4)). 

#### 2.2.2. rVE Across Different Influenza Seasons

The impacts of different seasonal influenza dynamics were evaluated by modelling three different season archetypes: 1. balanced co-circulation of A/H1N1 and A/H3N2, 2. A/H1N1 dominant season, and 3. A/H3N2 dominant season. Vaccination was assumed to occur in September, with protection beginning in October. The epidemic was modelled as a Gaussian distribution centered on January (the peak month), with a standard deviation σ = 1.5 months, consistent with the distribution of influenza activity observed in US sentinel surveillance over the past decade. The total seasonal attack rate was set at 7.5% among unvaccinated adults aged ≥65 years, based on data from a systematic review of 32 randomized controlled trials [26]. The monthly strain proportions underlying each archetype are shown in Supplementary Figure S1 [27]. A sensitivity analysis was performed varying the timing of vaccination and the epidemic peak, with two vaccination timing scenarios (September or October) and three epidemic peak months (December, January, or February) being considered for each of the three season archetypes.

#### 2.2.3. Estimation of Public Health Impacts

Coverage equivalence analysis was used to evaluate the level of vaccine HD-IIV4 coverage needed to match the case prevention rate achieved by mRNA-1010 at a reference coverage rate. For this analysis, we set the reference rate at 65%, based on current influenza vaccine coverage rates in adults ≥65 years of age in the US [28] and an eligible population size of 58 million. VE was estimated using the season-integrated model outlined above. The absolute risk difference between the two vaccines was calculated as the difference in the projected cumulative seasonal incidence of laboratory-confirmed influenza, and the number needed to vaccinate as its reciprocal. As the parametric bootstrap was applied to the monthly estimates, bounds on the season-integrated quantities were obtained by the propagation of the monthly intervals.

### 2.3. Ethics Statement

The P303 and P304 trials were approved by appropriate institutional review boards (IRBs) and were conducted according to the applicable principles of the International Council for Harmonization Good Clinical Practice guidelines, the E6(R2) Good Clinical Practice guidelines, the principles of the Declaration of Helsinki, and all local laws or regulations. The analysis presented is covered under the initial ethics approval for the P303 and P304 trials. This analysis was based on aggregate patient data from the mRNA-1010-P303 Part C and mRNA-1010-P304 trials. Moderna Inc. granted access and permission to reuse the datasets from the P303 Part C and P304 trials for this study. No additional institutional review board review was required for this analysis of the P303C and P304 trials.

## 3. Results

### 3.1. Calibration

Calibration of the model reproduced the rVE estimates from the P304 trial to within 0.1% of the original estimates, with an estimated rVE of 29.6% against A/H1N1 (observed rVE from the original trial: 29.6%), 22.2% against A/H3N2 (original: 22.2%), 29.1% against B/Victoria (original: 29.1%), and 29.5% across strains, confirming the numerical convergence of the calibration (original: 29.6%; Supplementary Table S4).

### 3.2. rVE of mRNA-1010 vs. HD-IIV4

Application of the model to estimate rVE of mRNA-1010 vs. HD-IIV4 in adults ≥65 years of age using data from the P303 trial demonstrated incremental benefits of the mRNA vaccine, with rVEs at Day 29 of 24.1% (18.2–29.5%) against A/H1N1, 14.5% (8.3–20.3%) against A/H3N2, and 21.0% (16.0–25.8%) against B/Victoria (Table 1).

**Figure 1:**
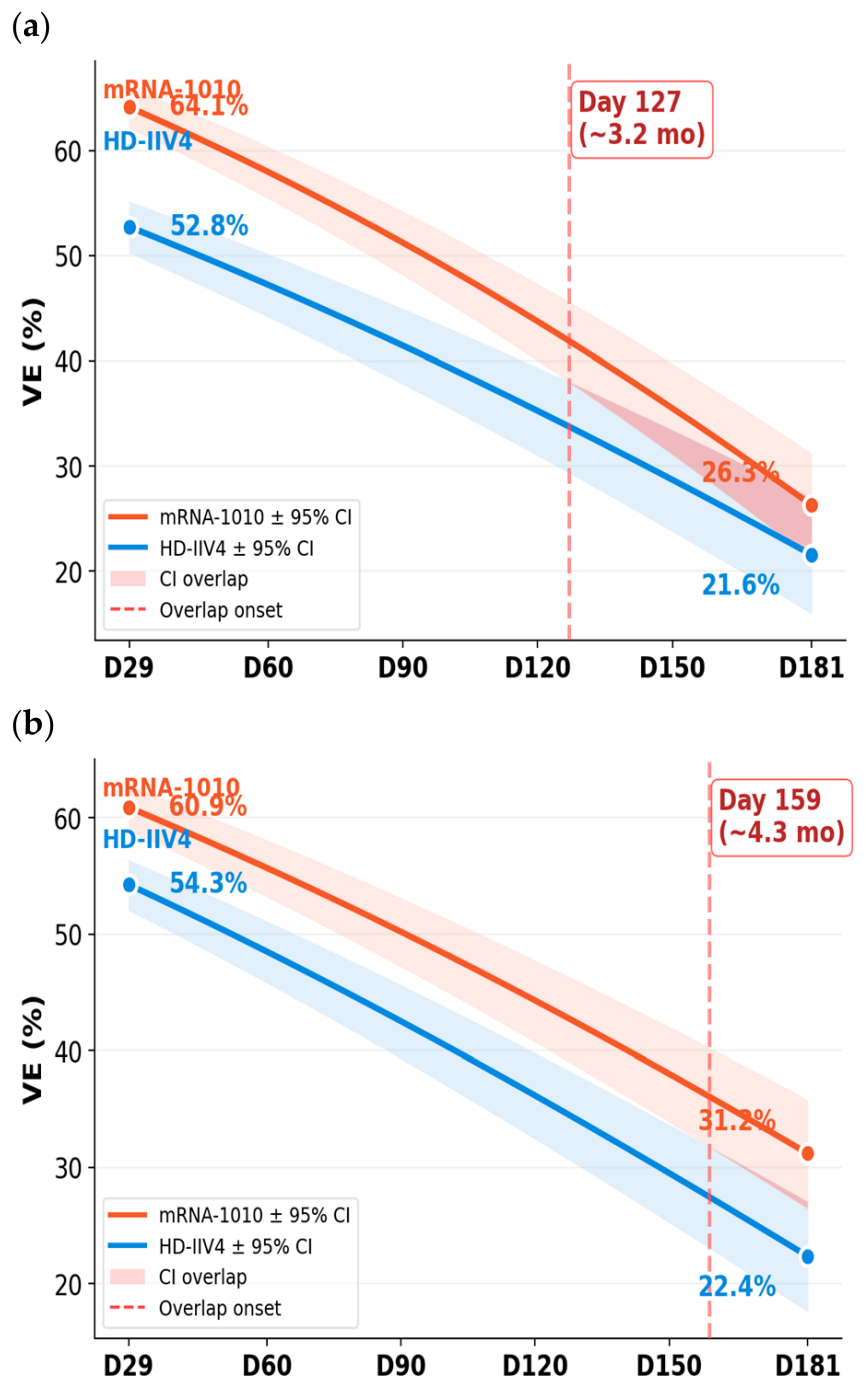

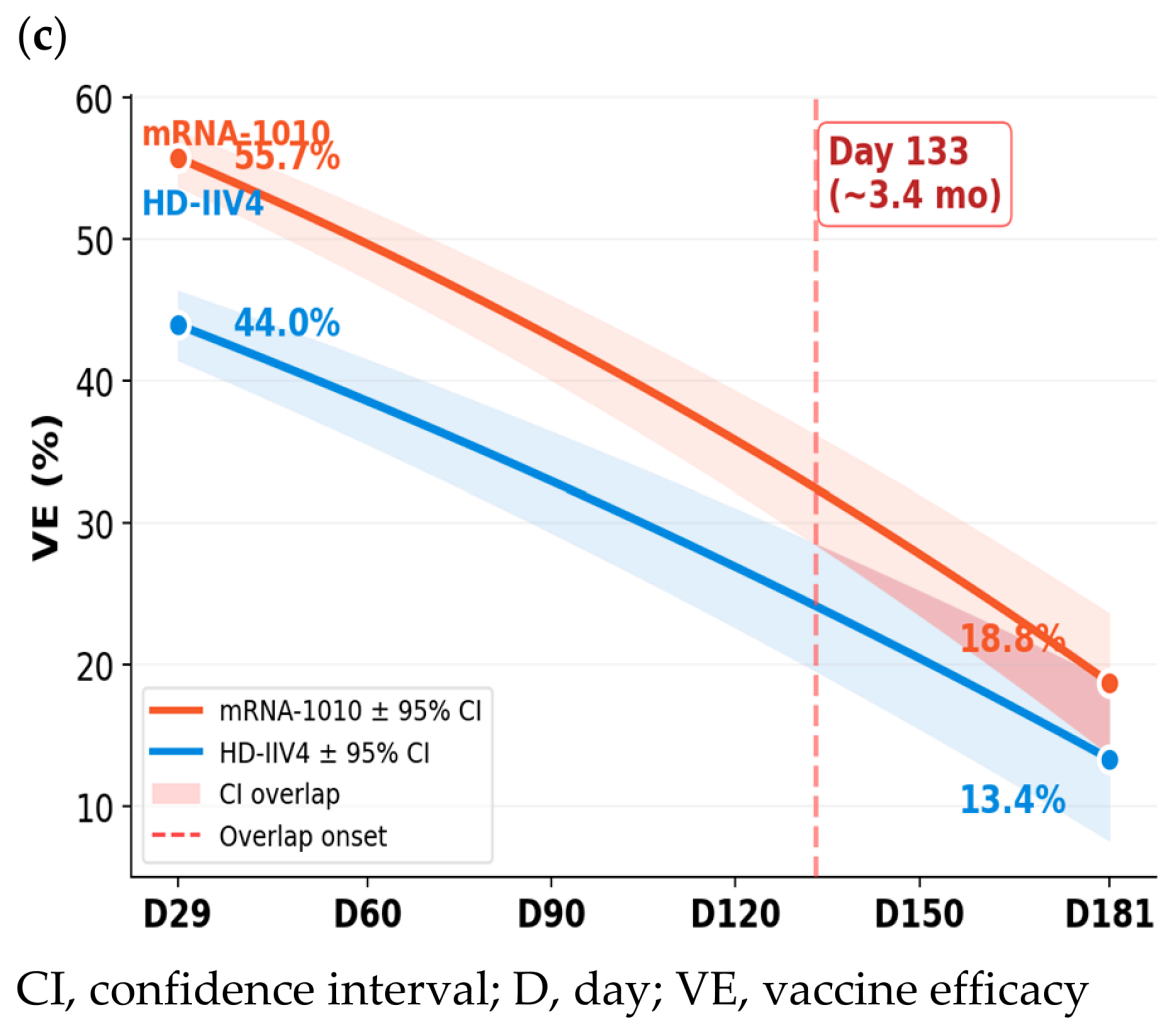
Estimated VE trajectory from Day 29 to Day 181 for mRNA-1010 and HD-IIV4 against (**a**) A/H1N1, (**b**) A/H3N2, and (**c**) B/Victoria, showing the day at which 95% CIs start to overlap for the two vaccines.

Evaluation of the continuous VE trajectory during the influenza season, accounting for waning VE over time from Day 29 through Day 181, showed that the 95% CIs of the VE estimate for each of the two vaccines started to overlap at Day 127 against A/H1N1, Day 159 against A/H3N2, and Day 133 against B/Victoria (Figure 1, Supplementary Table S5). Evaluation of the estimated rVE at time points from Day 29 to Day 181 showed the maintenance of positive point estimates of rVE after the CIs began to overlap, with point estimates of 6.0%, 11.4%, and 6.2% on Day 181 against A/H1N1, A/H3N2, and B/Victoria, respectively (Supplementary Table S6). Analysis of the degree of overlap of the 95% CIs in the period from overlap onset to Day 181 showed that true convergence (rVE <5% and CI overlap >50%) was not met against any of the influenza strains at any point during the evaluated period (Supplementary Table S7), as the rVE criterion was binding throughout: the projected rVE did not fall below 5% for any strain at any time point, the minimum being 6.0% for A/H1N1 at Day 181.

### 3.3. Season-Integrated rVE

**Figure 2:**
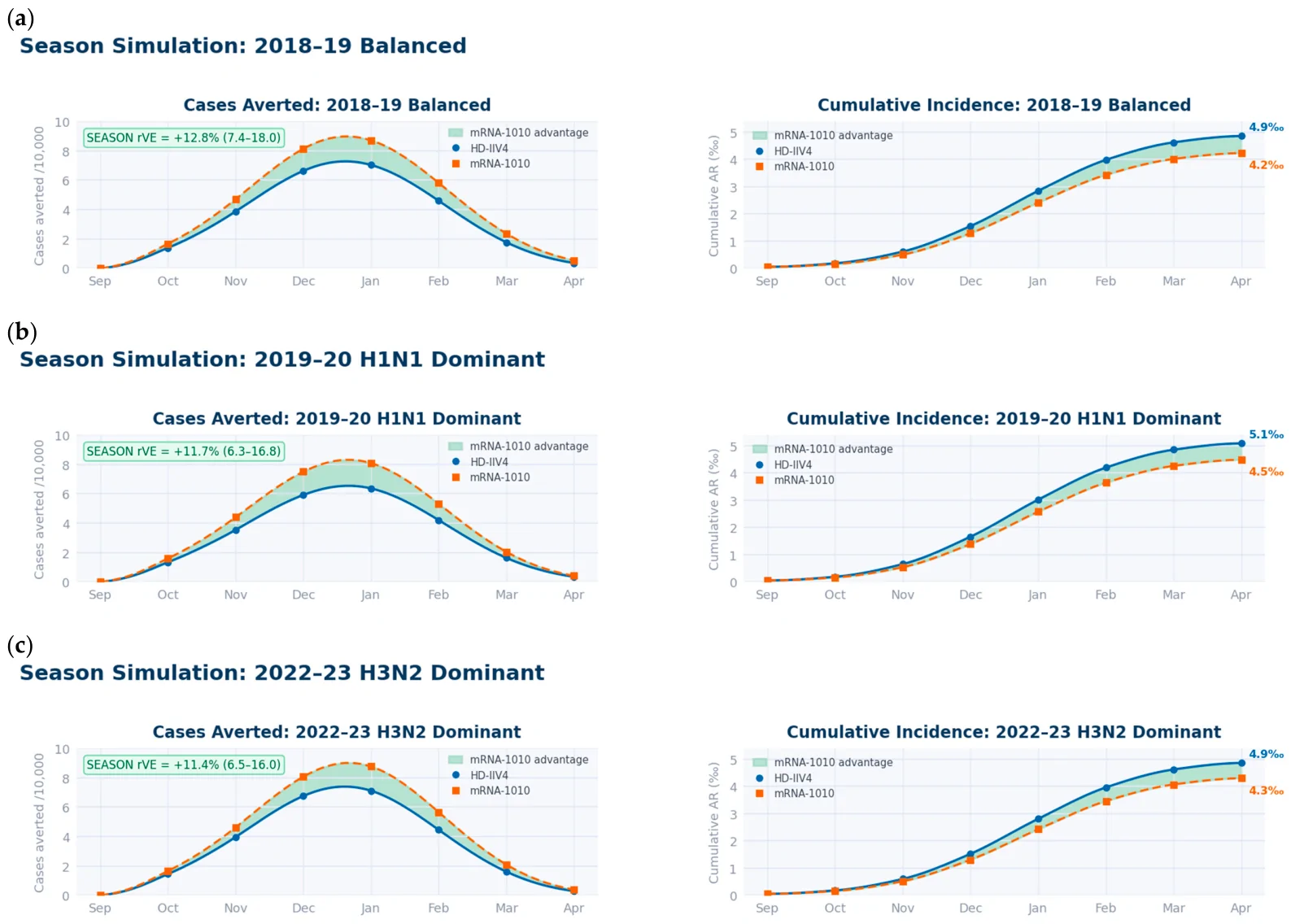
Cases averted per month and cumulative incidence of cases for (**a**) balanced season, (**b**) A/H1N1-dominant season, and (**c**) A/H3N2-dominant season.

Assessment of the season-integrated rVE showed a global rVE against all three strains of 12.4% (3.4–21.3%), with individual point estimates of 11.6% to 12.6% (Table 2). Estimation of the effects of varying the season archetype showed similar rVE estimates per month and for the entire season, with cross-strain estimates of 12.8% (7.4–18.0%) in a balanced season, 11.7% (6.3–16.8%) in an A/H1N1-dominant season, and 11.4% (6.5–16.0%) in an A/H3N2-dominant season (Table 3, Supplementary Table S8). The greatest concentration of cases averted by the use of mRNA-1010 instead of HD-IIV4 would occur in the peak transmission months for all three season archetypes, with smaller incremental benefits seen later in the season (Figure 2). Sensitivity analysis showed a consistent benefit of mRNA-1010 over HD-IIV4 across all 18 combinations of vaccination and peak incidence timing, with rVE estimates ranging from 8.8% to 14.3% (Supplementary Table S9). Changing the timing of the epidemic peak had the greatest effects on rVE estimates, with earlier peaks resulting in a higher rVE.

### 3.4. Public Health Impact

Coverage equivalence analysis showed that to match the case prevention achieved by mRNA-1010 at 65% coverage, HD-IIV4 would require a coverage of 79.2% to 81.7% across the three season archetypes evaluated (Table 4). This translates to needing to vaccinate an additional 8.2 to 9.7 million adults ≥65 years of age in the United States who are not currently being vaccinated. The corresponding absolute difference in the risk of laboratory-confirmed influenza was 6.3 (95% CI: 2.3–10.3) per 1000 vaccinees in a balanced season, 6.0 (2.0–10.1) per 1000 in an A/H1N1-dominant season, and 5.6 (2.0–9.2) per 1000 in an A/H3N2-dominant season, giving numbers needed to vaccinate of approximately 159 (97–430), 167 (99–513), and 180 (109–513) respectively.

## 4. Discussion

As no head-to-head studies have directly compared the efficacy of mRNA-1010 with HD-IIV4, we developed a correlate-of-protection model to evaluate the rVE of the two vaccines against laboratory-confirmed influenza, based on immunogenicity data in adults ≥65 years of age and efficacy data. mRNA-1010 was projected to have higher VE than HD-IIV4 across strains and broader strain coverage in all analyses performed, including at the time of peak immunity post-vaccination (Day 29), throughout the influenza season, and across the different types of influenza season archetypes. Overall, the use of HD-IIV4 would require ~9 million extra individuals ≥65 years of age to be vaccinated in the United States to achieve the same protection as mRNA-1010.

The projected season-integrated rVE of mRNA-1010 versus HD-IIV4, ranging from +11.4% to +12.8% depending on the season archetype, is driven by eliciting consistently higher antibody titers, with geometric mean ratios of approximately 1.2 to 1.3, despite HD-IIV4 having a four-fold higher antigen dose than standard-dose vaccine. This immunogenicity difference likely lies in the differences between the two platforms in the way antigen is presented to the immune system. mRNA-1010 delivers a lipid nanoparticle-encapsulated mRNA encoding the native hemagglutinin sequence directly into host cells [29]. The resulting intracellular expression of HA enables antigen presentation via both MHC class I and MHC class II pathways, activating CD8+ cytotoxic T cells and CD4+ T follicular helper cells. The latter are the principal drivers of germinal center reactions, where B cell affinity maturation occurs and high-affinity antibodies, reflected in high HAI titers, are produced [30]. In contrast, HD-IIV4, as an inactivated extracellular antigen, is processed primarily through the endosomal pathway and presented via MHC class II, with limited MHC class I cross-presentation. Comparative studies have shown that nucleoside-modified mRNA-LNP vaccines elicit substantially more potent T follicular helper and germinal center B cell responses than adjuvanted protein and inactivated virus vaccines [31]. Beyond antigen presentation, the mRNA molecule itself activates innate immune sensors including TLR7/8 and the cytosolic receptors RIG-I and MDA5, generating a type I interferon response that acts as an endogenous adjuvant [32]. HD-IIV4 is formulated without an adjuvant and does not activate these pathways [31,33]. Consequently, mRNA-1010 generates a higher-magnitude, higher-quality humoral response owing to a more efficient antigen delivery mechanism, resulting in greater immunogenicity and associated VE.

As the CoP model presented here is HAI-based, it only captures part of the humoral dimension of the immune response and does not take into account any differences in cellular and non-HA antibody responses. T cell responses have previously been shown to be a better correlate of protection from influenza vaccines in older adults than HAI titers [34], and pre-existing CD4+ T cell responses have been shown to protect against symptomatic influenza independently of antibody levels [35]. mRNA-1010 has been shown to induce a broader functional antibody profile, including antibody-dependent cellular cytotoxicity, complement activation, and phagocytosis, than inactivated influenza vaccines [36], as well as strong HA-specific CD4+ and CD8+ T cell responses [37], none of which are captured in the current analysis. The net effect of these omissions on the projected rVE cannot be determined. HD-IIV4 contains neuraminidase whereas mRNA-1010 encodes haemagglutinin alone, and yet the neuraminidase content of inactivated influenza vaccines is neither standardized nor routinely quantified, leaving its contribution unquantifiable. While a greater cell-mediated response to mRNA-1010 is likewise plausible in light of the responses reported for this platform [37], no comparative cellular immunogenicity data are available for P303 Part C.

Beyond the intrinsic platform advantages of mRNA versus inactivated influenza vaccines, our framework assumes a perfect antigenic match between vaccine strains and circulating viruses, which is unlikely in real-world situations where a degree of mismatch is expected, particularly for egg-based vaccines. Egg adaptation results in HAI antibodies that are measurable using standard assays but are optimized against egg-adapted epitopes rather than epitopes on the circulating viruses, likely resulting in poorer VE [10,38]. In seasons where egg adaptation occurs, egg-based HD-IIV4 would have reduced effective immunogenicity while mRNA-1010, which encodes the WHO reference virus sequence, would be unaffected. In these seasons, it is therefore likely that our framework would further underestimate the rVE, although the degree of underestimation would be dependent on the specific season dynamics.

The coverage equivalence analysis translates the projected rVE into population-level terms relevant to payers and public health decision-makers. To match the case prevention achieved by mRNA-1010 at 65% coverage, HD-IIV4 would require 79–82% coverage—an increase of 14–17 percentage points, representing 8–10 million additional vaccinations in the ≥65-year-old population in the US, and considerably above the WHO and CDC target coverage rates of 75% and 70%, respectively [39,40]. Influenza vaccination coverage among US adults ≥65 years of age has remained in the 65–70% range for over a decade despite sustained public health campaigns, financial incentives, pharmacist-administered vaccination, and reminder systems [41]. The structural barriers to coverage improvement, including vaccine hesitancy, access inequities, and disengagement from preventive care, have proven resistant to intervention, and therefore it is unlikely that a coverage rate of 80% in older adults is regularly achievable. The practical implication of switching from HD-IIV4 to mRNA-1010 represents a more achievable path to reducing the residual influenza burden in older adults than further efforts to increase coverage.

As the results of this study were based on a modelled framework rather than observed efficacy results, there are a number of limitations. Firstly, β and N50 were calibrated using P304 data and applied to P303 GMTs to project the rVE against HD-IIV4, an approach that assumed that the sigmoidal relationship between HAI titer and protection observed in P304 is transportable to the P303 population. GMTs from P303 and P304 support this assumption as observed titers were on a consistent numerical scale despite the two studies using different vaccine formulations and dosing regimens (e.g., A/H3N2 GMTs differed by only 1.8% between P303 Part A ≥ 65 years of age and P304 ≥ 50 years of age). Similarly, we assumed an N50 of 40. This value derives from early challenge studies in younger adults [20] but has been confirmed using individual-level data in adults ≥65 years and older [19]. While older adults produce lower GMT levels post-vaccination compared to younger populations, reflecting reduced antibody production capacity due to immunosenescence, the antibody level required to achieve 50% protection remains constant at approximately 40 [19]. While enhanced vaccine formulations (high-dose, adjuvanted, recombinant) for older adults are designed to overcome reduced production capacity and push the host’s antibody concentration above the N50 threshold, they do not change the threshold itself [8,42]; therefore, the use of this value for this age group was appropriate for our framework. Neither external nor internal out-of-sample validation was available: the only efficacy trial in adults ≥65 years of age with a published titer–protection curve is that of Dunning et al., which is also the source of the model form used here, and the arm-specific Day 29 GMTs for the ≥65-year subgroup of P304 are not reported. Secondly, the analysis was based on the use of population-level GMTs rather than individual antibody titers. As protection is a non-linear function of the titer, protection evaluated at the group geometric mean is not identical to the mean of individual protection probabilities. Since β was calibrated on aggregate GMTs to reproduce an observed rVE, any bias from this approximation is absorbed into the calibrated value, which is therefore an effective parameter conditional on the aggregate-GMT formulation. The reported confidence intervals were obtained by a parametric bootstrap on the P303 Part C GMTs, sampling each arm from the standard error of its geometric mean titer. They therefore quantify the uncertainty on the population estimate of VE, which is the quantity of interest for a between-arm comparison, rather than the variability of individual titers. This differs in kind from the exploratory strain-specific intervals of P304, which reflect sampling variability in observed case counts, and accounts for the contrast seen for B/Victoria: the P304 interval is wide because few B cases were observed, whereas the projected interval reflects the precision with which the input GMTs were themselves estimated. The intervals remain conditional on β, λ, and N50, which were not treated as random quantities. Thirdly, we assumed an efficacy waning rate of 7% per month, which was applied symmetrically to both vaccines at the calibration stage. This symmetry was not an assumption of equal waning but was a deliberate design choice ensuring that any difference in projected rVE derived exclusively from the difference in Day 29 GMTs, not from a modelled waning advantage for either platform. The assumption of similar waning was supported by mRNA-1010 antibody titers at individual timepoints remaining higher than HD-IIV4 titers throughout follow-up, confirmed by GMRs > 1 at all measured points. It should also be noted that the current model was based solely on HAI responses, and did not include other immune responses such as NA or cellular responses, which may differ between the vaccine platforms and thus further contribute to rVE. Finally, it could be interpreted that the overlapping 95% CIs from Day 127 for A/H1N1, Day 133 for B/Victoria, and Day 159 for A/H3N2 could be an indication of the equivalence of the vaccines from this point onwards. We performed a power analysis to further estimate the effects of this convergence, with none of the rVE estimates meeting the criteria for true convergence, although technically the estimates were no longer statistically different. Nevertheless, it should be noted that the majority of cases (~79%) had already occurred prior to these timepoints, which should be taken into account when evaluating the clinical significance of the converging confidence intervals. The model was also applied to HD-IIV4 immunogenicity data only, and does not establish a direct comparison of the performance of mRNA-1010 relative to adjuvanted or recombinant enhanced vaccines, although this relationship could potentially be inferred indirectly though further comparisons of HD-IIV4 with other vaccine types. While VE and rVE are efficacy estimates by design, since both P303 and P304 are randomized controlled trials, the projections presented here are expected to approximate real-world effectiveness: as noted above, neither trial was conducted in a season of documented egg adaptation or significant antigenic mismatch, the factors most consistently associated with divergence between efficacy and effectiveness for influenza vaccines. Existing evidence suggests that this convergence remains an expectation rather than a demonstrated equivalence, since other determinants of real-world effectiveness—uncontrolled administration conditions, broader comorbidity profiles, and adherence—are not captured by a randomized trial design; however, more research is needed in this area.

## 5. Conclusions

In summary, in this CoP model based on head-to-head immunogenicity data, mRNA-1010 was projected to have a higher VE than HD-IIV4 across a wide range of epidemiological conditions in adults ≥65 years of age. This incremental benefit was driven by an intrinsic mRNA-platform immunological benefit rather than by any particular season or strain configuration, with a significantly positive rVE vs. HD-IIV4 in all season archetypes evaluated. Overall, this correlate-of-protection framework provides a reproducible, transparent analytical foundation for comparing mRNA-1010 against HD-IIV4.

## Figures and Tables

**Table 1:** Projected VE at Day 29 for mRNA-1010 and HD-IIV4 in adults ≥65 years of age.

Strain	VE mRNA-1010 (95% CI)	VE HD-IIV4 (95% CI)	rVE mRNA-1010 vs. HD-IIV4 (95% CI)
A/H1N1	64.1% (62.1–66.1)	52.8% (50.3–55.2)	+24.1% (18.2–29.5)
A/H3N2	60.9% (59.0–62.9)	54.3% (52.1–56.4)	+14.5% (8.3–20.3)
B/Victoria	56.1% (54.1–58.0)	44.4% (41.9–46.8)	+21.0% (16.0–25.8)

CI, confidence interval; rVE, relative vaccine efficacy; VE, vaccine efficacy.

**Table 2:** Season-integrated VE and rVE, weighted by monthly case distribution from P304.

Strain	VE HD-IIV4	VE mRNA-1010	rVE (95% CI)	Weight ^a^
A/H1N1	34.4%	42.5%	+12.4%	50%
A/H3N2	35.4%	43.6%	+12.6%	44%
B/Victoria	26.6%	35.1%	+11.6%	6%
Global (weighted)	—	—	+12.4% (3.4–21.3%)	—

rVE, relative vaccine efficacy; VE, vaccine efficacy, ^a^ Global rVE weighted by strain proportions (H1N1: 50%, H3N2: 44%, B: 6%). Bounds derived by propagation of the monthly bootstrap intervals (N = 5000).

**Table 3:** Seasonal simulation results for different season archetypes for mRNA-1010 versus HD-IIV4.

Season Archetype	VE HD-IIV4 (95% CI)	VE mRNA-1010 (95% CI)	rVE (95% CI)	Cases Averted per 1000 (95% CI)
Balanced	34.3% (31.4–37.1)	42.7% (40.2–45.1)	+12.8% (7.4–18.0)	6.3 (2.3–10.3)
A/H1N1-dominant	31.2% (28.3–34.1)	39.2% (36.7–41.8)	+11.7% (6.3–16.8)	6.0 (2.0–10.1)
A/H3N2-dominant	34.3% (31.8–36.8)	41.7% (39.4–44.0)	+11.4% (6.5–16.0)	5.6 (2.0–9.2)

rVE, relative vaccine efficacy; VE, vaccine efficacy.

**Table 4:** Coverage equivalence analysis estimating the required coverage of HD-IIV4 to match the number of cases averted by mRNA-1010 at a coverage level of 65%.

Season Archetype	VE mRNA-1010	VE HD-IIV4	HD-IIV4 Coverage Required	Additional Coverage Required	Additional Vaccinations Required
Balanced	42.7%	34.3%	80.9%	+15.9%	9.2 million
A/H1N1-dominant	39.2%	31.2%	81.7%	+16.7%	9.7 million
A/H3N2-dominant	41.7%	34.3%	79.2%	+14.2%	8.2 million

VE, vaccine efficacy.

## Data Availability

The data that support the findings of this study are available from the corresponding author upon reasonable request.

## Supplementary Materials

The following supporting information can be downloaded at: https://www.mdpi.com/article/10.3390/vaccines14090750/s1, Equation (S1): The Sigmoidal Protection Curve; Equation (S2): Incremental Vaccine Efficacy; Equation (S3): Waned VE at Timepoint t; Equation (S4): Season-Integrated VE and rVE; Supplementary Table S1: Input GMTs at Day 29 and Day 181 with 95% confidence intervals (adults ≥65 years) from the P303 study comparing immunogenicity of mRNA-1010 vs. HD-IIV4; Supplementary Table S2: Decision rules for evaluating the impact of overlapping VE confidence intervals on equivalence of VE estimates between the two vaccines; Supplementary Table S3: Distribution of cases over the influenza season in the P304 study; Supplementary Table S4: Calibration of the model against the reported P304 VE data; Supplementary Table S5: Estimated day of onset of overlap of 95% confidence intervals surrounding the VE estimate based on GMT observations at Day 29 and Day 181 from the P303 study in adults ≥65 years; Supplementary Table S6: Estimated VE and rVE with 95% CI at key timepoints from Day 29 to Day 181 based on data from P303 in older adults ≥65 years; Supplementary Table S7: Evaluation of impact of CI overlap for VE estimates for mRNA-1010 vs. HD-IIV4 in the period from the onset of overlap through Day 181; Supplementary Table S8: Monthly effective VE weighted by strain for balanced, A/H1N1-dominant, and A/H3N2-dominant seasons; Supplementary Table S9: Sensitivity analysis estimating rVE across 18 combinations of vaccination timing, epidemic peak month, and season archetype; Supplementary Figure S1: Monthly strain distribution by season archetype.
