# Supplementary material for: Estimating the Relative Vaccine Efficacy of mRNA-1010 to High-Dose Influenza Vaccine in Adults ≥65 Years Using a Correlate of Protection Framework
Source: Vaccines (Basel). 2026 Aug 28;14(9):750. doi: 10.3390/vaccines14090750 (PMC13611724; doi:10.3390/vaccines14090750)
Supplement: Supplementary file 1 [file vaccines-14-00750-s001.zip › vaccines-4494632-supplementary.pdf]

Supplementary Materials

Equation S1. The Sigmoidal Protection Curve

The probability of protection π at a given HAI titre G is expressed as:

$$\pi(G) = 1 / (1 + \exp(-\beta \times \ln(G / N50)))$$

where N50 = 40 is the titre at which protection probability equals 50%, and β is the strain-specific slope parameter calibrated on P304 data. The natural logarithm ensures that the model operates on the multiplicative titre scale, consistent with the log-normal distribution of HAI titres in population samples.

Equation S2. Incremental Vaccine Efficacy

VE is expressed as the fraction of preventable cases actually prevented by vaccination, relative to pre-existing baseline immunity:

$$VE\_D29 = (\pi(G\_post) - \pi(G\_pre)) / (1 - \pi(G\_pre))$$

where G_post is the post-vaccination GMT at Day 29 and G_pre is the pre-vaccination baseline GMT. The denominator $(1 - \pi(G\_pre))$ represents the susceptibility remaining after pre-existing immunity; the numerator represents the additional protection contributed by vaccination.

Equation S3. Waned VE at Timepoint t

Applying the Ferdinands linear waning model symmetrically to both arms:

$$VE(t) = \max(0, VE\_D29 - \lambda \times t)$$

where t is time in months after Day 29 and $\lambda = 0.07$ per month. The function is truncated at zero to prevent negative VE values at late timepoints under extreme waning scenarios.

Equation S4. Season-Integrated VE and rVE

The season-integrated VE is the weighted average of waned VE values at each temporal bin midpoint:

$$VE\_int = \Sigma\_k (w\_k \times VE(t\_k))$$

where w_k are the temporal weights (3.1%, 15.3%, 28.6%, 31.6%, 17.7%, 3.3%, 0.4%) and t_k are the bin midpoints (0.75, 1.5, 2.5, 3.5, 4.5, 5.5, 6.5 months post-vaccination). The relative vaccine efficacy is derived from the Cox proportional-hazards approximation:

$$HR = (1 - VE\_mRNA\_int) / (1 - VE\_comparator\_int)$$

$$rVE = 1 - HR = 1 - (1 - VE\_mRNA\_int) / (1 - VE\_comparator\_int)$$

This formulation compares the residual risks of the two arms and is equivalent to 1 minus the hazard ratio under proportional hazards.

Supplementary Table S1. Input haemagglutination inhibition (HAI) GMTs at Day 29 and Day 181 with 95% confidence intervals (adults ≥65 years) from the P303 study comparing immunogenicity of mRNA-1010 vs HD-IIV4 [1].

Strain	Arm	Pre-vaccination GMT	GMT Day 29 (95% CI)	GMT Day 181 (95% CI)
**A/H1N1**	HD-IIV4	50.02	130.17 (122.94–137.83)	70.84 (64.52–77.78)
	mRNA-1010	50.02	174.72 (165.06–184.94)	76.84 (70.37–83.90)
**A/H3N2**	HD-IIV4	34.19	116.30 (109.52–123.49)	54.60 (49.37–60.39)
	mRNA-1010	34.19	141.35 (133.06–150.16)	66.12 (59.60–73.35)
**B/Victoria**	HD-IIV4	104.57	196.23 (187.68–205.18)	124.06 (115.42–133.36)
	mRNA-1010	104.57	247.96 (237.27–259.12)	133.11 (124.48–142.33)

CI, confidence interval; GMT, geometric mean haemagglutination inhibition (HAI) titre, measured against a common cell-derived antigen panel in each trial (see Methods); HD-IIV4, high-dose quadrivalent inactivated influenza vaccine

Supplementary Table S2. Decision rules for evaluating the impact of overlapping VE confidence intervals on equivalence of VE estimates between the two vaccines

Classification	CI overlap	rVE point estimate	Interpretation
**Insufficient power**	<15%	≥10%	Real difference may persist; overlap reflects precision limit only
**Near-threshold**	15–30%	8–10%	Borderline — approaching but not crossing clinical threshold
**Moderate grey zone**	30–50%	5–8%	Signal weakening but positive
**True convergence**	>50%	<5%	Equivalence of vaccines

CI, confidence interval; rVE, relative vaccine efficacy. Classification is determined by the rVE criterion; CI overlap is reported as descriptive context and was not binding for any classification reported in this analysis.

Supplementary Table S3. Distribution of cases over the influenza season in the P304 study

Time after vaccination	Percentage of cases
14 days to 1 month	3.1%
>1 month to 2 months	15.3%
>2 months to 3 months	28.6%
>3 months to 4 months	31.6%
>4 months to 5 months	17.7%
>5 months to 6 months	3.3%
>6 months to end of influenza season	0.4%

Supplementary Table S4. Calibration of the model against the reported P304 VE data

Strain	β	VE Day 29 SD-IIV4[a]	VE Day 29 mRNA-1010[a]	VE integrated model SD-IIV4[b]	VE integrated model mRNA-1010[b]	rVE model	rVE observed
**A/H1N1**	1.1425	41.4%	65.1%	19.8%	43.6%	29.6%	29.6%
**A/H3N2**	1.0437	48.0%	64.3%	26.5%	42.8%	22.2%	22.2%
**B/Victoria**	1.1504	42.3%	65.4%	20.8%	43.8%	29.1%	29.1%
**Global[c]**	—	—	—	—	—	26.5%	26.6%

SD-IIV4, standard-dose quadrivalent inactivated influenza vaccine; VE, vaccine efficacy

[a]VE estimated at Day 29 based on sigmoidal protection curve where N50 = 40 and β as stated in table

[b]VE estimate taking into account intraseason waning (λ = 7%/month)[2] and temporal weighting by case distribution.

[c]Global rVE weighted by strain proportions (H1N1: 50%, H3N2: 44%, B: 6%).

Supplementary Table S5. Estimated day of onset of overlap of 95% confidence intervals surrounding the VE estimate based on GMT observations at Day 29 and Day 181 from the P303 study in adults ≥65 years

Strain	Arm	GMT Day 29 (95% CI)	GMT Day 181 (95% CI)	VE CI overlap onset
**A/H1N1**	HD-IIV4	130.17 (122.94–137.83)	70.84 (64.52–77.78)	Day 127
	mRNA-1010	174.72 (165.06–184.94)	76.84 (70.37–83.90)	
**A/H3N2**	HD-IIV4	116.30 (109.52–123.49)	54.60 (49.37–60.39)	Day 159
	mRNA-1010	141.35 (133.06–150.16)	66.12 (59.60–73.35)	
**B/Victoria**	HD-IIV4	196.23 (187.68–205.18)	124.06 (115.42–133.36)	Day 133
	mRNA-1010	247.96 (237.27–259.12)	133.11 (124.48–142.33)	

CI, confidence interval; GMT, geometric mean titre; HD-IIV4, high-dose quadrivalent inactivated influenza vaccine; VE, vaccine efficacy

Supplementary Table S6. Estimated VE and rVE with 95% CI at key timepoints from Day 29 to Day 181 based on data from P303 in older adults ≥65 years.

Day	VE HD-IIV4 (95% CI)	VE mRNA-1010 (95% CI)	rVE (95% CI)	CI status
**A/H1N1**				
Day 29	52.8% (50.3–55.1)	64.1% (62.1–66.1)	24.1% (18.2–29.5)	Separated
Day 59	47.3% (45.0–49.6)	58.1% (56.1–59.9)	20.4% (15.3–25.2)	Separated
Day 90	41.5% (38.9–43.9)	51.3% (49.1–53.3)	16.7% (11.4–21.6)	Separated
Day 120	35.2% (31.9–38.4)	43.7% (40.9–46.3)	13.1% (6.7–18.9)	Separated
Day 127	— Overlap onset —			
Day 150	28.5% (24.2–32.7)	35.3% (31.4–38.9)	9.5% (−3.5–16.6)	Overlap
Day 181	21.5% (15.8–27.0)	26.2% (21.0–31.0)	6.0% (−3.8–14.5)	Overlap
**A/H3N2**				
Day 29	54.3% (52.1–56.4)	60.9% (59.0–62.9)	14.5% (8.3–20.3)	Separated
Day 59	48.7% (46.6–50.7)	55.8% (54.0–57.6)	14.0% (8.8–18.8)	Separated
Day 90	42.6% (40.3–44.9)	50.3% (48.1–52.2)	13.4% (8.3–18.2)	Separated
Day 120	36.2% (33.3–39.0)	44.3% (41.5–46.9)	12.7% (6.8–18.3)	Separated
Day 150	29.4% (25.6–33.2)	37.9% (34.3–41.5)	12.1% (5.0–18.7)	Separated
Day 159	— Overlap onset —			
Day 181	22.4% (17.6–27.3)	31.2% (26.5–35.8)	11.4% (−0.8–19.2)	Overlap
**B/Victoria**				
Day 29	44.4% (41.9–46.8)	56.1% (54.1–58.0)	21.0% (16.0–25.8)	Separated
Day 59	39.1% (36.9–41.4)	50.2% (48.3–52.1)	18.1% (13.7–22.3)	Separated
Day 90	33.5% (30.9–36.0)	43.6% (41.4–45.6)	15.1% (10.6–19.6)	Separated
Day 120	27.4% (24.2–30.6)	36.2% (33.5–38.9)	12.2% (6.7–17.3)	Separated
Day 133	— Overlap onset —			
Day 150	20.9% (16.6–25.1)	28.2% (24.4–31.8)	9.2% (−2.4–15.7)	Overlap
Day 181	14.0% (8.3–19.4)	19.3% (14.3–24.2)	6.2% (−3.1–14.1)	Overlap

CI, confidence interval; HD-IIV4, high-dose quadrivalent inactivated influenza vaccine; rVE, relative vaccine efficacy; VE, vaccine efficacy

Supplementary Table S7. Evaluation of impact of CI overlap for VE estimates for mRNA-1010 vs HD-IIV4 in the period from the onset of overlap through Day 181

Strain	Period	rVE central	GMR (mRNA-1010 vs HD-IIV4)	CI overlap	Classification
A/H1N1	Day 127–141	10.6–12.3%	1.15–1.17	0.3–17.1%	Insufficient power
	Day 147–162	8.2–9.9%	1.11–1.14	23.8–39.6%	Near-threshold
	Day 169–181	6.0–7.4%	1.09–1.10	46.6%*	Moderate grey zone
A/H3N2	Day 159–181	11.4–11.9%	1.21	0.2–6.5%	Insufficient power
B/Victoria	Day 133–142	10.2–10.9%	1.12–1.13	1.1–7.9%	Insufficient power
	Day 143–161	8.2–9.9%	1.09–1.12	13.0–32.8%	Near-threshold
	Day 168–181	6.2–7.5%	1.07–1.09	40.0%*	Moderate grey zone

CI, confidence interval; GMR, geometric mean ratio; HD-IIV4, high-dose quadrivalent inactivated influenza vaccine; rVE, relative vaccine efficacy. Ranges reflect the values computed at the reported timepoints within each period (Supplementary Table 5). *Overlap continues to increase through Day 181; the corresponding rVE at Day 181 (6.0% for A/H1N1, 6.2% for B/Victoria) is reported in Supplementary Table 6.

Supplementary Table S8. Monthly effective VE weighted by strain for balanced, A/H1N1-dominant, and A/H3N2-dominant seasons

Season archetype	Month	VE HD-IIV4 (95% CI)	VE mRNA-1010 (95% CI)	rVE (95% CI)
Balanced	October	51.9% (50.2–53.5)	62.3% (60.9–63.6)	21.6% (17.6–25.4)
	November	47.3% (44.8–49.6)	57.4% (55.4–59.3)	19.2% (13.8–24.3)
	December	41.5% (38.6–44.3)	50.9% (48.4–53.3)	16.1% (10.0–21.9)
	January	35.2% (32.0–38.4)	43.6% (40.8–46.3)	13.0% (6.6–19.1)
	February	28.8% (25.6–31.8)	36.4% (33.4–39.1)	10.7% (5.1–16.1)
	March	21.6% (18.2–24.9)	28.8% (25.6–32.0)	9.2% (3.5–14.7)
	April	13.6% (10.0–17.2)	20.1% (16.5–23.5)	7.5% (−1.9–13.0)
	Season rVE			**12.8% (7.4–18.0%)**
A/H1N1-dominant	October	49.8% (48.5–51.3)	59.6% (58.4–60.8)	19.5% (16.1–22.7)
	November	43.2% (41.3–45.2)	53.7% (52.1–55.3)	18.5% (14.3–22.3)
	December	37.0% (34.6–39.5)	46.9% (44.8–49.0)	15.7% (10.8–20.2)
	January	31.9% (29.1–34.8)	40.5% (37.8–43.0)	12.6% (7.2–17.7)
	February	26.3% (22.8–29.9)	33.3% (30.0–36.5)	9.5% (3.0–15.3)
	March	20.0% (15.8–24.4)	25.0% (20.9–29.0)	6.3% (−1.0–13.0)
	April	13.2% (7.9–18.7)	15.6% (10.4–20.6)	2.8% (−6.0–10.8)
	Season rVE			**11.7% (6.3–16.8%)**
A/H3N2-dominant	October	53.9% (52.1–55.5)	61.6% (60.0–63.2)	16.7% (12.2–21.1)
	November	48.4% (46.2–50.5)	56.4% (54.4–58.3)	15.5% (10.3–20.3)
	December	42.3% (39.7–44.7)	50.5% (48.2–52.8)	14.2% (8.7–19.4)
	January	35.6% (32.8–38.3)	43.9% (41.3–46.5)	12.9% (7.3–18.1)
	February	27.9% (24.9–30.7)	35.4% (32.7–38.1)	10.4% (5.1–15.6)
	March	19.6% (16.3–23.0)	25.5% (22.5–28.6)	7.3% (1.8–12.8)
	April	10.9% (6.9–15.0)	14.4% (10.7–18.1)	3.9% (−2.3–9.8)
	Season rVE			**11.4% (6.5–16.0%)**

CI, confidence interval; HD-IIV4, high-dose quadrivalent inactivated influenza vaccine; rVE, relative vaccine efficacy; VE, vaccine efficacy

Supplementary Table S9. Sensitivity analysis estimating rVE across 18 combinations of vaccination timing, epidemic peak month, and season archetype

Vaccination	Peak	Balanced	A/H1N1-dominant	A/H3N2-dominant
September	December	+13.8%	+13.2%	+13.1%
September	January	+12.8%	+11.7%	+11.4%
September	February	+11.6%	+10.5%	+10.3%
October	December	+14.3%	+13.6%	+13.5%
October	January	+13.1%	+12.1%	+11.8%
October	February	+9.8%	+8.8%	+9.1%

rVE, relative vaccine efficacy

Supplementary Figure S1. Monthly strain distribution by season archetype [3] Proportions were derived from WHO FluNet US sentinel surveillance data covering June 2016 to February 2026. For each month, the number of specimens positive for A/H1N1pdm09, A/H3 and B was expressed as a proportion of all subtyped positives, with influenza A specimens not subtyped reallocated in proportion to the subtyped A specimens of the same month. Seasons were assigned by specimen collection date. Three archetypes were defined from these data: a balanced season with co-circulation of A/H1N1 and A/H3N2, an A/H1N1-dominant season, and an A/H3N2-dominant season. These monthly proportions determine the strain weighting applied to the projected VE at each timepoint in the seasonal simulations reported in Table 3 and Supplementary Table 8 of the main manuscript.

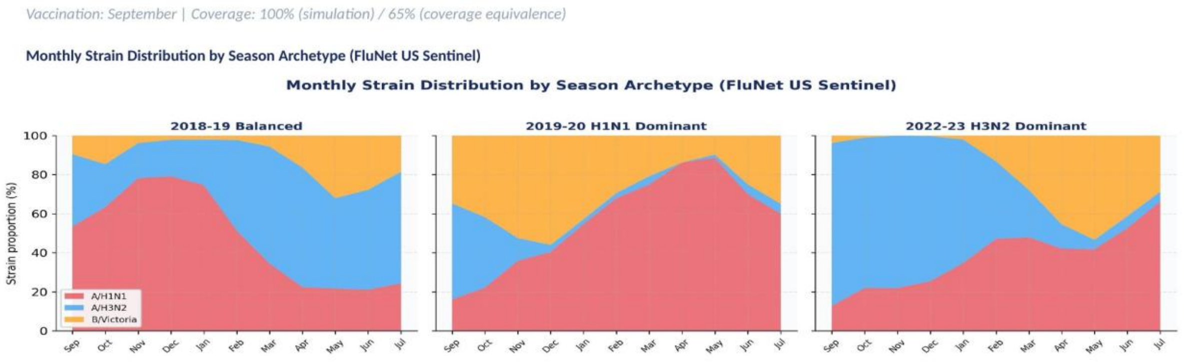

References

1. Soens, M.; Ananworanich, J.; Hicks, B.; Lucas, K.J.; Cardona, J.; Sher, L.; Livermore, G.; Schaefers, K.; Henry, C.; Choi, A.; et al. A phase 3 randomized safety and immunogenicity trial of mRNA-1010 seasonal influenza vaccine in adults. *Vaccine* **2025**, *50*, 126847, doi:10.1016/j.vaccine.2025.126847.
2. Ferdinands, J.M.; Fry, A.M.; Reynolds, S.; Petrie, J.; Flannery, B.; Jackson, M.L.; Belongia, E.A. Intraseason waning of influenza vaccine protection: Evidence from the US Influenza Vaccine Effectiveness Network, 2011-12 through 2014-15. *Clin Infect Dis* **2017**, *64*, 544-550, doi:10.1093/cid/ciw816.
3. World Health Organization. FluNet, Global Influenza Surveillance and Response System (GISRS). US Sentinel surveillance data, June 2016 – February 2026. Available online: https://www.who.int/tools/flunet (accessed on 15 April 2026).
